# Pharmacist-led vaccination services in the Middle East

**DOI:** 10.1186/s40545-023-00664-8

**Published:** 2023-12-29

**Authors:** Faten Naif Aldajani, Mohammed Aldosari

**Affiliations:** Pharmacy Department, Alnakheel Medical Center, Al Mahdyah, 7222 Riyadh, Saudi Arabia

## Abstract

Successful Vaccine uptake relies heavily on the effectiveness of vaccination services. Expanding the scope of pharmacists’ involvement in vaccination services can significantly improve vaccination coverage. The level of pharmacists’ engagement in immunization services varies globally. The aim of this paper is to describe the current role of pharmacists in vaccination services in the Middle Eastern countries. The provision of vaccination services by pharmacists in the region has evolved notably in recent years. The extent of pharmacists’ involvement in immunization services varies from one country to another in the region. They play a more active role in the delivery of vaccinations, not only facilitating but also administering vaccines. Future studies on pharmacist-led vaccination services in these countries are necessary to assess the value of the expanded practice of pharmacists in this field, especially due to the scarcity of evidence.

## Introduction

Vaccine uptake has contributed to the eradication of many vaccine-preventable diseases and the reduction of their incidence worldwide. According to the World Health Organization (WHO), approximately 2 to 3 million lives can be saved each year by vaccination [1–3]. Successful Vaccine uptake depends on the effectiveness of vaccination services, which encompass the entire process from vaccine distribution to education and administration. These services are vital for protecting individuals and communities against vaccine preventable diseases.

Typically, most of the vaccination services are provided by physicians or nurses in primary care clinics or hospitals, while pharmacists’ role is limited to vaccine distribution [4]. In 1996, the American Pharmacist Association (APhA) adopted a policy to encourage pharmacist involvement in the vaccination program, which mainly consist of three levels to increase immunization coverage. First, the pharmacist can be an advocate and raise public awareness of the benefits of vaccination uptake. Second, the pharmacist can facilitate the accessibility of vaccines in the community pharmacy by hosting other health care providers, such as physicians or nurses in the pharmacy setting to administer vaccines. Third, the pharmacist can be a vaccine administer or immunizer after completing the required training and obtaining legal permission from the government [5].

Expanding pharmacists’ scope to be engaged in vaccination services can positively influence vaccination coverage. Isenor et al. found that the immunization rate improved when pharmacists were involved in immunization services, regardless of their role [4]. For example, as one of the strategies employed during the H1N1 pandemic in 2009 was to use community pharmacies as “point of dispensing (POD)” to deliver the pandemic vaccine to the public, recognizing the valuable role of the pharmacist as a legally authorized immunizer [6]. Many patients were comfortable receiving the H1N1 vaccine from pharmacists in the pharmacy setting, considering it a preferred and convenient place for immunization. For another instance, in the United States and some of European countries, pharmacists were involved in Coronavirus disease 2019 (COVID-19) vaccination services including vaccine administration to broaden (COVID-19) vaccines accessibility [7, 8]. These pandemics brought pharmacists to the forefront and underscored the importance of their involvement in all aspects of vaccine services [9].

The extent of pharmacist engagement in immunization services varies worldwide. In the United states and several European countries, pharmacists have a long-standing history of involvement in vaccine distribution and administration [3, 10]. Moreover, in some countries, such as Argentina, Australia, Canada, the Philippines, Portugal and South Africa, pharmacists are legally authorized to administer vaccines. In other countries, such as China and Malaysia, pharmacists are mainly involved in vaccine advocacy [10]. Meanwhile, in Russia, pharmacist play a role in vaccination advocacy [10]. Pharmacists have yet to become involved in any vaccination services in some countries, such as Ukraine and Paraguay [10]. In the Middle Eastern countries, the evidence regarding the pharmacist's role in vaccination services is limited.

## Objectives

The purpose of this paper is to describe the pharmacist’s current role in vaccination services in the Middle East countries.

## Pharmacist-led vaccination services in the Middle East

In Middle Eastern countries, pharmacists are primarily involved in controlling inventory, ordering and distributing vaccines. The level of pharmacists’ involvement in vaccination services varies across the region. The majority of Middle Eastern countries participating in the International Pharmaceutical Federation (FIP) survey reported that pharmacists’ roles in their countries are primarily focused on vaccine advocacy [6]. Further details about these countries are discussed in this paper. The selection of all these countries was based on the criteria of WHO’s Eastern Mediterranean region [11]. The pharmacists’ role in vaccination services in these countries is summarized in Table 1.

**Table 1:** Description of pharmacist’ role in vaccination services in the Middle Eastern countries

Country	Role of pharmacist in vaccination services
Iraq [10]	Pharmacists are involved in vaccine advocacy
Israel [20]	Pharmacists are involved in vaccine advocacy, vaccine facilitation and vaccine administration
Jordan [23]	Pharmacists are involved in vaccine advocacy, vaccine facilitation and vaccine administration
KSA [26, 27]	The pharmacist involved in vaccine advocacy, vaccine facilitation and vaccine administration
Lebanon [10, 29]	Pharmacists are involved in vaccine facilitation
Pakistan [10]	Pharmacists are involved in vaccine advocacy and vaccine facilitation
Turkey [38]	Pharmacists are involved in vaccine advocacy, vaccine facilitation and vaccine administration
UAE [10, 40]	Pharmacists are involved in supplying, stocking, distributing and administering vaccines

### Iraq

Iraq, with a population of 32 million who served by almost 11,000 licensed pharmacists, has a ratio of one pharmacist for every 2909 people, as reported by Human Resources for Health [12, 13]. Pharmacists in Iraq practices both traditional and clinical pharmacy in the public and private sectors [14]. However, based on the results of a 2016 survey by FIP, the role of the pharmacists in immunization services in Iraq primarily involves vaccination advocacy. In addition, vaccination coverage among children in Iraq was reported to be low in 2015, as estimated by the WHO and the United Nations Children’s Fund (UNICEF). The data revealed that the coverage for the third dose of diphtheria–tetanus–pertussis vaccine, oral polio vaccine, and the first dose of measles—containing vaccine stood at 58%, 63%, and 57%, respectively. These figures fall below the national coverage target (95%) [15, 16].

Furthermore, the rate of full immunization for infants during their first year is 34% in Erbil and Duhok [17]. Nearly 50% of the factors contributing to the low immunization coverage in Duhok are related to the lack of vaccine education. Numerous studies worldwide have demonstrated the positive impact of vaccine education in increasing immunization rates [17]. Therefore, expanding the scope of pharmacy practice to involve pharmacists in delivering immunization services can have a positive impact on vaccination rates.

### Israel

There is an approximate ratio of one pharmacist for every 1228 people, based on a population of 8.6 million, who are served by nearly 7000 licensed pharmacists [18, 19]. In addition to the traditional role of the pharmacists as many countries, pharmacists in Israel play a role as advocates. They educate the public about the importance of Influenza vaccine. Moreover, formal training in vaccine administration is mandatory for both undergraduate and postgraduate students in pharmacy schools [20]. Overall, there is a demand to broaden immunization services led by pharmacists in all community pharmacies in Israel.

### Jordan

In Jordan, with an estimated population of 11.3 million, there were a total of 26,000 registered pharmacists in December 2020 [21, 22]. Pharmacists role in Jordan regarding vaccination services was limited to vaccine advocacy and counseling to the public [23]. As outcome of the initiatives undertaken by the Jordanian Pharmacists Association (JPA), in 2020, the Ministry of health granted permission for pharmacists to administer seasonal influenza vaccines at community pharmacy settings. This approval marked a broadening of the responsibilities and scope for community pharmacists. [23]

### Kingdom of Saudi Arabia (KSA)

The ratio of licensed pharmacists to the population in this region is approximately 1 pharmacist per 1167 people, based on a population of 36 million who served by 30,840 pharmacists. The scope of pharmacy practice has evolved from a drug-oriented focus to a patient-oriented approach while still maintaining the pharmacist’s traditional roles as the foundation of practice [24, 25].

Pharmacists in Saudi Arabia often play a role in educating the public about the importance of vaccinations [26]. In 2019, the amended executive regulation of the health proficiency Law granted community pharmacists the authority to deliver immunization services, including vaccine administration [26]. Presently, Community pharmacists in the region are permitted to administer Influenza vaccines to adults [27].

During the COVID-19 pandemic, the Saudi ministry of health established a collaborative agreement with various community pharmacies. They played a significant role in administering COVID-19 vaccines as part of the national vaccination campaign [26]. The provision of immunization services in community pharmacies is still in the early stages of development, reflecting a positive shift toward the broader role of pharmacists in public health and preventive care. The participation of pharmacists in immunization services in Saudi Arabia aligns with global trends aimed at increasing vaccination coverage and improving public health outcomes. This expanded role not only helps individuals access essential vaccines more conveniently but also contributes to the nation's overall health and well-being.

### Lebanon

The pharmacist-to-population correlation in Lebanon is approximately 0.0799% for a population of 4 million served by 3198 licensed pharmacists. More than half of these licensed pharmacists work in community pharmacies [28]. Pharmacy practice in Lebanon extends beyond the primary scope of pharmacists. They facilitate immunization delivery to the public by hosting authorized healthcare providers, such as physicians or nurses who can administer vaccines, specifically the flu vaccine, in community pharmacies. However, pharmacists tend not to actively promote immunization uptake due to the absence of policies or guidelines encouraging their involvement at this level. In addition, pharmacists are not authorized to administer vaccines, because they have not received formal vaccination training [10, 29]. In the literature, the vaccination rate among Lebanese ambulatory adult population falls below the standard level [30]. Lack of awareness regarding the importance of regular influenza vaccination is considered a factor leading to limited vaccination uptake. This emphasizes the need to advocate for taking the influenza vaccine by increasing the public’s knowledge of its efficacy and safety [30]. Integrating the full potential of the pharmacist's role in immunization services in community pharmacies can significantly contribute to public health [10].

### Pakistan

The Islamic Republic of Pakistan has a pharmacist-to-population ratio of nearly 0.0045%, with an estimated population of 220 million and a total of 10,000 licensed pharmacists according to the Pakistan Pharmacy Council in 2010 [31–33]. The role of pharmacists in Pakistan is typically revolves around drug dispensing, distribution and storage. In general, pharmacy practice in this country significantly lags behind many other countries worldwide. There is a deficiency in pharmacy services across all sectors, primarily due to a shortage of pharmacists, a lack of public recognition of the pharmacy profession, minimal government interest in the pharmacy profession and a shortage of human resources [34]. In addition, most community pharmacies are operated by non-professional personnel who lack basic pharmacy knowledge and skills. This situation results in limited public interaction with community pharmacists [31, 35]. However, the authorization for immunization services in community pharmacies is granted to government vaccinators, physician or nurses. While most vaccine administrations typically occur in hospitals or clinics, pharmacists play a role in immunization advocacy by distributing vaccine leaflets and participating in immunization advisory committee. They educate people about immunization and provide vaccine information. Nevertheless, they are still not permitted to administer vaccines to the public. [10]. Expanding pharmacy practice is essential to clarify the role pharmacists in immunization [36].

### Turkey

Turkey, with an estimated population of 84 million, has pharmacists who play a crucial role in immunization [37]. They are responsible for vaccine storage in pharmacies and are legally permitted to administer vaccines to the public at community pharmacies [38]. Similar to many other countries, pharmacists also serve as vaccine educators as part of pharmacy-led immunization efforts to increase the immunization rate. Their multifaceted role in vaccine education, facilitation, administration, storage and safety monitoring demonstrates the importance of pharmacists in advancing public health through immunization services.

### The United Arab Emirates (UAE)

In the UAE, there is an approximate ratio of one pharmacist for every 1135 people, based on a population of 9 million, who are served by nearly 7930 licensed pharmacists [39]. Previously, pharmacists’ involvement in vaccination services revolved around supply, stocking and distribution of vaccines [10, 40]. However, with recent drug legislation in the UAE, Pharmacists have expanded their role to administer vaccines following adequate training [41]. This transition enables pharmacists to take on a more active role in public health by contributing to the prevention of diseases through accessible vaccination services.

## Discussion

Evidence of the current role of pharmacists in vaccination services in Middle Eastern countries remains limited, despite the valuable role played by pharmacists worldwide in improving vaccination accessibility and increasing immunization coverage. However, small number of studies in these countries have been identified and the pharmacists’ role in vaccination programs have been highlighted.

Typically, the initial step for pharmacists to engage in immunization services is through training. Pharmacists involvement in vaccination training courses is crucial as it forms the cornerstone of providing appropriate vaccination services to the public. Training course represent the initial step taken in developed countries, where the role of pharmacists in vaccination programs has significantly evolved. This evolution ranges from the foundational vaccination advocacy to the highest level of vaccination program involvement, vaccination administration.

A training program necessitate a robust infrastructure with well-trained staff to enhance pharmacists’ knowledge and skills in immunization, enabling them to deliver appropriate vaccination services. Pharmacist can educates the public on the importance of immunization (vaccination advocacy), facilitates disease prevention strategies (vaccination facilitation) and administers vaccine to eligible individuals (vaccine administration).

The level of Pharmacists’ involvement in vaccination services varies across all countries in the region. Based on this review, pharmacists in Middle Eastern countries were traditionally involved in vaccine storage, distribution and advocacy. However, recently, the role of pharmacists has expanded in some regional countries to include involvement in vaccine facilitation and administration.

The most frequently reported role of pharmacists is vaccine advocacy, involving raising public awareness about the significance of vaccine uptake to diminish barriers, such as vaccine refusal and delays in acceptance. Individual acceptance of immunization is often influenced by the ease, accessibility and convenience of reaching vaccination settings. Hence, integrating community pharmacies as immunization settings can enhance public immunization acceptance in Middle Eastern countries. Furthermore, the facilitation of vaccine delivery in community pharmacies may alleviate distance-related barriers for some individuals in accessing vaccines. In addition, the avoidance of contact with patients having infectious diseases in the waiting rooms of primary clinics is considered one of the potential benefits of using community pharmacies as a vaccination setting.

Including pharmacists as vaccine administer in vaccination program can have a positive impact on vaccination coverage across the entire population in the region. Various studies have reported an increase in immunization rate when pharmacists are integrated into vaccination programs to serve as vaccine administer [42]. Based on this review, it has been noticed that the flu vaccine for adults is the most commonly delivered and administered vaccine to the eligible population in community pharmacy settings in some Middle Eastern countries. However, the administration of other types of vaccines in community pharmacy settings, either by pharmacists or other health care providers, has not been identified. Moreover, the facilitation of delivering childhood vaccines in community pharmacies for children, along with the administration of these vaccines by pharmacists in these settings, has not been thoroughly studied. Further research is required to address the gaps in the existing literature.

Some limitations on this paper have been noted. First, most relevant studies have not deeply described the pharmacists’ role in vaccination services in the Middle Eastern countries, which may not accurately reflect the actual state of pharmacist practices concerning vaccination in this region. Second, due to the scarcity of published literature, there is difficulty in clearly delineating the extent of pharmacists’ participation at each level of vaccination programs. Finally, the absence of documentation of pharmacists’ efforts in immunization services in the Middle Eastern countries renders the role of pharmacists unremarkable.

## Conclusion

Recently, there has been a notable expansion in the role of pharmacists in vaccination services across countries in the region. The extent of pharmacists’ involvement in immunization services varies from one country to another. Most of the countries studied in the region, pharmacists play a more active role in the delivery of vaccinations, not only facilitating but also administering vaccines. The involvement of pharmacists in vaccination services aims to enhance public health by promoting and improving immunization coverage across the Middle East. Future studies on pharmacist-led vaccination services in these countries are necessary to assess the value of pharmacists’ expanded practices in this area, given the scarcity of evidence.

## Data Availability

Not applicable.

## Abbreviations

WHO: World Health Organization
APhA: American Pharmacist Association
POD: Point of dispensing
ACPE: Accreditation Council for Pharmacy Education
HHS: Health and Human Services
US: United States
PREP Act: Public Readiness and Emergency Preparedness Act
COVID-19: Coronavirus disease 2019
FIP: International Pharmaceutical Federation
UNICEF: United Nations Children’s Fund
KSA: Kingdom of Saudi Arabia
UAE: United Arab Emirates
GCC: Gulf Cooperation Council
MOH: Ministry of Health
ADR: Adverse Drug Reaction
DHA: Dubai Health Authority
JPA: Jordanian Pharmacists Association
